# Economic Burden of Drug Use in Patients with Acute Burns: Experience in a Developing Country

**DOI:** 10.1155/2009/734712

**Published:** 2009-08-31

**Authors:** Kolawole Olubunmi Ogundipe, Ismaila Abiona Adigun, Babatunde Akeeb Solagberu

**Affiliations:** ^1^Division of Plastic and Reconstructive Surgery, Department of Surgery, University of Ilorin Teaching Hospital, 240001 Ilorin, Nigeria; ^2^Division of Orthopaedics and Trauma Surgery, Department of Surgery, University of Ilorin Teaching Hospital, 240001 Ilorin, Nigeria

## Abstract

*Background/Objective*. Burn injury is a devastating injury. The economic drain on the patient's purse is equally devastating. Few studies have examined the cost of managing burn patients particularly the drug component. *Methods*. The financial implication of drug use in the management of 69 consecutive patients admitted by the burn unit over a period of two years was retrospectively analysed. *Results*. Thirty-six (52.2%) patients were males and 33 (47.8%) females with a mean age of 17.9 years (SD = 18.4). The patients spent an average sum of $91.21 to procure drugs; 84.3% of the costs were for antibiotics, 11.1% for analgesics, and 4.6% for others. *Conclusion*. Significant amount of money is spent on the procurement of drugs. Most of the money is spent on prescribed antibiotics. Measures that reduce antibiotics use in burn management might relief patients of the huge economic burden associated with its use.

## 1. Introduction

Burn injury is one of the most devastating injuries anyone can sustain and remain alive. Masellis considered it to be the most complex trauma which can strike a human organism [1]. The mortality following burn injuries used to be very high, but improvement in management has resulted in increased survival of the burnt patient over the years [2, 3]. This reduction in mortality has been attributed to the establishments of burn centres and changes in burn wound treatment policy [4]. With increasing survival comes a huge economic drain on the patient's pulse which is comparable with the resultant dis ment, disability, and emotional instability following the injury as the overall cost of care of burnt patients is very high [5]. 

Studies have been conducted to support the need for surgical interventions in decreasing the cost of care and to show the profitability of burn centres [6–8]. These studies have shown that when burnt patients are managed in burn centres where surgical interventions, namely early excision and wound cover, are carried out, the length of hospital stay is reduced significantly. Few studies however have examined the cost associated with burn patients' care, still fewer researches have reviewed the pattern of drug utilization and the cost of the medications in the management of burns [9, 10]. This study was done to determine the pattern of drugs utilization and cost of drugs in the management of patients with acute burns in a tertiary hospital in a developing country.

## 2. Patients and Method

A retrospective analysis was conducted based on the case reports of 69 consecutive patients that presented to the accident and emergency (A and E) ward of our hospital, a tertiary institution located in North Central region of Nigeria, with acute burns and admitted by the plastic unit from the 1st of April 2003 to the 31st of March 2005. Their treatment sheets and drug charts were examined to determine the types of drugs prescribed and administered, their doses and duration of use. The age, sex, percentage burnt surface area and depth, length of hospital stay, and disposition were determined for each patient. The mode of treatment, namely: conservative versus surgical intervention, as well as the types of dressings carried out was noted. The medications used were classified into pharmacological classes {antibiotics, analgesics, sedatives, tetanus prophylaxis, antacids and anti-ulcer regimen, and accessories for drug administration (infusion giving sets, syringes and needles, canulae, etc.)} according to the national drug formulary. The cost of the drugs was calculated in accordance with the hospital pharmacy acquisition cost [USD] of similar drugs in recent time (2006). The medications that were administered in the operating theatre for patients that underwent operative procedures were excluded from the analysis. The results are presented in simple tables and charts using the Microsoft Excel 2003 Software.

## 3. Result

**Figure 1:**
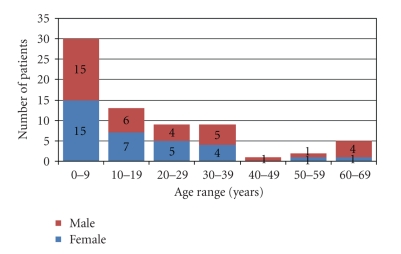
Sex and age distribution of patients.

**Figure 2:**
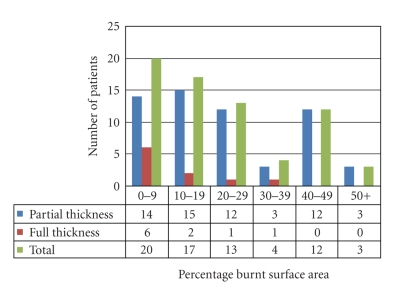
Frequency of total burnt surface area and depth of burn.

Of the 69 burn patients that were admitted during the period under review, 36 (52.2%) were male and 33 (47.8%) were females giving a male to female ratio of 1.1 : 1. Though the mean age was 17.9 years with a standard deviation of 18.4years (range 1 month to 68 years), 30 (43.5%) of the patients were less than 10 years old, and only 7 (10.1%) were 50 years and above (Figure 1). Twenty (29.0%), 17 (24.7%), 13 (18.8%), 4 (5.8%), 12 (17.4%) and 3 (4.3%) patients had burns involving 0–9, 10–19, 20–29, 30–39, 40–49, 50 and above percent burnt surface area (BSA) respectively (Figure 2). The mean body surface area burnt was 21.5%. The burns were partial thickness in 59 (85.5%) and full thickness in 10 (14.5%) patients. 

**Figure 3:**
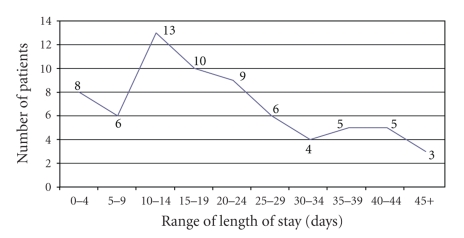
Length of hospital stay.

Figure 3 shows the length of hospital stay, with an average stay of 15.4 days (range 1–74 days). Forty-four (63.8%) patients were discharged while 18 (26.1%) died and 7 (10.1%) left against medical advice. Eight (44.4%) of the deaths occur in less than 10 days of admission. Most of the patients were managed conservatively. This involved the application of topical dressings that were changed initially daily and subsequently on alternate day or longer period as dictated by the wound condition. The main materials used for dressing include honey in 48 patients, sulfatulle or its variants in 28 patients, dermazine (1% silver sulfadiazine) in 31 patients and antibiotic impregnated Vaseline gauze in 24 patients. Most of the patients actually had a combination of the dressing materials used at various periods. The choice of the dressing materials was largely determined by the patients' clinical presentation as well as their financial status. Some forms of operative interventions, including escharotomies and skin grafting, were performed in 7 (10.1%) patients. The plastic surgery unit did not have a dedicated theatre as at the time of management of the patients presented. All patients presenting to the unit and requiring surgery had to be operated on a single weekly list that was highly competitive. Emergencies also had to compete for the limited space available to all surgical specialities.

On an average the cost of management (dressings, surgery, drugs, admission charges and nursing care) of a patient was about $274.56 (range $87.92–1029.23). This exclude indirect cost to the patients incurred on things like transportation, feeding, as well as cost of disability, and work days lost. Table 1 illustrate the cost incurred by the patients in procuring the drugs administered. An average sum of $91.21 (with a range of $13.42–420.86) was spent per patient; 84.3% of the costs were for antibiotics, 11.1% for analgesics, and 4.6% for others. This also amount to an average sum of $5.92 per day of admission per patient (average length of hospital stay is 15.4 days) and $4.25 per percent burnt surface area (mean BSA 21.5%).

## 4. Discussion

The management of burn injuries still poses a serious challenge. Burn centres, where they are available have reduced the menace of the injury. Adigun and Abdulrahman [11] had earlier stressed the need to create burn centres in Nigeria, today a number of burn centres exist in the countries. We still do not have a burn centre in our hospital though. The almost equal gender distribution found in this study is different from the male predominance reported in other studies within this country [12, 13]. Children below the age of 10 years constitute 43.5% of patients in this study, followed by the age range 10–19 years (18.8%). A similar study by Olabanji et al. [14] also showed that 53% of the studied patients were in their first decade of life.

The mean TBSA of 21.5% obtained in this study was low compared to two previous studies at Ibadan, Nigeria where mean TBSA of 36% and 38% were obtained four years apart [15]. This difference in mean TBSA might account for the lower mortality rate of 26.1% obtained in this study as against the mortality rate of 36% and 34% in the Ibadan studies. Fifty (72.5%) patients sustained burn injury less than 30% TBSA, 85.5% of which was partial thickness. This may in a way be responsible for the 15.4 days average length of hospital stay noted in this study.

As noted majority of the burns are <30% TBSA (<15.5% of these full thickness) and mostly scald in paediatric age group. Little wonder that very few patients had any operative intervention. Most of these patients were managed by initial debridement under conscious sedation followed by a course of antibiotics, analgesic and routine dressings (usually honey, dermazine or antibiotics impregnated Vaseline gause). Some of the patients that would have benefited from split skin grafts to enable early wound healing could not have the procedure because of lack of fund. Limited theatre space also contributed significantly to the low rate of operative intervention carried out. It is had to convince the theatre staff and fellow surgeons that a patient who sustained burn requires emergency operation in the face of other general surgical emergencies. This could only be reverted only when there is a burn unit with its own theatre and dedicated staff. Studies had shown that early excision and skin grafting of burn (and other operative interventions) not only reduces mortality, but also reduces the length of hospital days and invariably the cost of treatment [16–18]. Since reducing the cost of injury without significantly affecting the quality of care given is the utmost desire of health care practitioners, burn practitioners should continue the campaign for the establishment of burn centres in the practice. de Roche after a study of the cost of burn care noted that the cost of care is extremely high and concluded that any economic effort for primary burn treatment , however high it may be , is justified if the duration of rehabilitation and invalidity can be reduced [19].

Lofts in his study noted an expenditure of $647 per patient per day or $927 per % burn for the total cost of a successful inpatient management of a major burn [18]. Burn care is expensive to procure, and this much more so in the developing countries where the average daily income per person is less than $1 (GDP of $320 per annual—World Bank Statistic 2003), and health insurance scheme was almost non-existent until recently. For a patient in such a poverty–ridden country to spend between $87.92 and $1029.23on burn care on a 15.4 day average is extremely high. There is a need to look inward to ways of making the cost of care cheaper for the patients. To the best of our knowledge, drug procurement is one the cheapest expenditure in the management of burn, most expenses are spent of on dressings, admission and nursing charges, surgical procedures and other miscellaneous expenses. The drugs, most especially antibiotics, also ought to be administered only when needed. In this study, on an average, a whooping $91.21 (33.2% of average total cost of care) was spent per patient in procuring drugs alone, this translates to $5.92 per day of admission per patient or $4.25 per patient per percent burnt surface area. It should be noted that most of the patients in this study were children, meaning that for an adult patient the cost spent could actually be far above this as the dosing regime in adult is higher than that in children. Most of the money (84.3%) was spent on prescribed antibiotics. Even though the use of antibiotics in burn management is controversial [20], usually we use a course of a cephalosporin or quilonone and metronidazole for our patients. Will it then be justifiable to withhold the prescription of antibiotics in order to minimize this high economic burden? Where well equipped burn centres with easily accessible auxiliary services such as the laboratories are available, a delay in the prescription of antibiotics may be justified in as much as early diagnosis and management of suspected infection are feasible. Though our centre is a tertiary institution, we do not have a burn centre; our patients could not readily provide finances for laboratory investigation and surgical interventions, preventing and monitoring for infection therefore become difficult. Thus one cannot shy away from the use of antibiotics if it remains the sure way of ensuring survival of the patient. Further studies are however needed to compare the cost of antibiotics use in the conservative setting we adopted with that incurred when early surgical interventions are carried out. Regardless of the finding, improved funding of patients' care will ensure that they get the appropriate treatment as at when due. This will reduce the morbidities incurred (wound infection, increased length of hospital stay, etc.) when treatments are delayed.

## 5. Conclusion

The money spent on drugs is enormous when compared with our available income. In as much as the best treatment is the cheapest, what chance then do we have as practitioners in the developing countries to curtail the cost of care of our burn patients! One cannot overemphasis the need for increased funding such as the extension of health insurance scheme to cover burn management. Few operative procedures were also carried out in this study group mostly because the patients could not afford them, it is expected that when funds are available for more operative interventions, the overall length of hospital stay will reduce, with an attending reduction in drug use and thus cost of money spent in their procurement. A comprehensive analysis of the total cost of burn care will also bring to the fore areas where cost may be curtailed without affecting the quality of the given care adversely.

## Figures and Tables

**Table 1:** Cost of drug use in management.

Prescribed group of drugs	Average cost of acquisition per patient ($*)	Range of drug acquisition cost per patient ($*)	% of total cost for drugs
Antibiotics	76.87	7.29–358.21	84.3
Analgesics	10.12	0.04–57.43	11.1
Sedatives	0.64	0–2.15	0.7
Tetanus prophylaxis	0.72	0.72	0.8
Anti-ulcer regime	0.70	0–4.80	0.8
Accessories	2.14	2.01–2.42	2.3

Total cost for all drugs	**91.21**	**13.42–420.86**	**100.0**

*United State Dollar.
